# Mechanical Stretch Increases MMP-2 Production in Vascular Smooth Muscle Cells via Activation of PDGFR-β/Akt Signaling Pathway

**DOI:** 10.1371/journal.pone.0070437

**Published:** 2013-08-07

**Authors:** Kyo Won Seo, Seung Jin Lee, Yun Hak Kim, Jin Ung Bae, So Youn Park, Sun Sik Bae, Chi Dae Kim

**Affiliations:** Department of Pharmacology, School of Medicine, and Medical Research Center for Ischemic Tissue Regeneration, Pusan National University, Yangsan, Gyeongnam, Republic of Korea; Cleveland Clinic Lerner Research Institute, United States of America

## Abstract

Increased blood pressure, leading to mechanical stress on vascular smooth muscle cells (VSMC), is a known risk factor for vascular remodeling via increased activity of matrix metalloproteinase (MMP) within the vascular wall. This study aimed to identify cell surface mechanoreceptors and intracellular signaling pathways that influence VSMC to produce MMP in response to mechanical stretch (MS). When VSMC was stimulated with MS (0–10% strain, 60 cycles/min), both production and gelatinolytic activity of MMP-2, but not MMP-9, were increased in a force-dependent manner. MS-enhanced MMP-2 expression and activity were inhibited by molecular inhibition of Akt using Akt siRNA as well as by PI3K/Akt inhibitors, LY293002 and AI, but not by MAPK inhibitors such as PD98059, SP600125 and SB203580. MS also increased Akt phosphorylation in VSMC, which was attenuated by AG1295, a PDGF receptor (PDGFR) inhibitor, but not by inhibitors for other receptor tyrosine kinase including EGF, IGF, and FGF receptors. Although MS activated PDGFR-α as well as PDGFR-β in VSMC, MS-induced Akt phosphorylation was inhibited by molecular deletion of PDGFR-β using siRNA, but not by inhibition of PDGFR-α. Collectively, our data indicate that MS induces MMP-2 production in VSMC via activation of Akt pathway, that is mediated by activation of PDGFR-β signaling pathways.

## Introduction

Excess hemodynamic forces, leading to mechanical stretch (MS) in VSMC, play an important role in vascular remodeling and atherosclerotic lesion formation [1], [2]. The complex process of vascular remodeling involves enhanced collagen decomposition and extracellular matrix reorganization [3]. These processes are regulated by the enzymatic activity of matrix metalloproteinases (MMP) within the vascular wall [4], [5]. In arteriovenous fistula and vein bypass graft model, MMP-2 and MMP-9 are overexpressed at the site of neointima after 2 wks of exposure to arterial pressure [6], [7]. Moreover, MMP-2 expression in VSMC is significantly increased in vulnerable regions of atherosclerotic plaques [8], [9], suggesting a pathogenic role for MMP-2 in the progression of plaque rupture in hypertension-related atherosclerosis.

Regulation of MMP activity may occur at multiple levels either by gene transcription and synthesis of inactive proenzymes, post translational activation of proenzymes, or via the interaction of secreted MMP with their inhibitors named tissue inhibitors of metalloproteinases [10]. All members of the MMP family are secreted by cells as inactive proenzymes that must be proteolytically processed to become activated. Besides enzymatic activation by other proteases [11], Akt signaling pathways are known to enhance MMP expression and activity *in vitro* study [12], [13]. Thus, activation of the Akt signaling pathway is probably required for MMP production in VSMC under MS.

MS activates epidermal growth factor (EGF) receptor in keratinocytes [14], and stimulates proliferation of VSMC via the insulin-like growth factor (IGF) receptor [15] and platelet-derived growth (PDGF) receptor [16], with the latter implicated in MS-induced embryonic stem cell differentiation into VSMC [17]. Among various growth factors, PDGF is the most potent VSMC mitogen released by platelets, endothelial cells, VSMC and many other cells at the site of injury [18]. The role of PDGF in the pathogenesis of arterial injury disorders, including atherosclerosis and post-angioplasty restenosis, has also been well established [19]. However, the individual role of PDGF isoforms in the pathogenesis of vascular remodeling in arterial hypertension has not been clarified.

Although receptor tyrosine kinases including receptors for EGF, FGF, IGF and PDGF have been proposed as mechanoreceptors in a variety of tissues [20], [21], it is still unclear whether these receptor tyrosine kinases play pivotal roles in the proximal mechanotransduction response of VSMC to mechanical stress. Thus, we identified cell surface mechanoreceptors that influence VSMC to produce MMP in response to MS. In addition, the cross-talk between responsible membrane receptors for MS and intracellular signaling pathways involved in MMP production was assessed.

## Methods

### Ethics statement and Animals

All animal procedures conformed with the Guide for the Care and Use of Laboratory Animals published by the US National Institute of Health (NIH Publication No. 85–23, revised 1996), and experimental protocols were approved by the Pusan National University Institutional Animal Care and Use Committee.

### Chemicals and Antibodies

Various signal pathway inhibitors and growth factor receptor inhibitors were purchased from Calbiochem (La Jolla, CA). Gelatin was obtained from Sigma (St. Louis, MO). MMP-2, PDGFR-α, -β, Akt, MAPK antibodies and phosphospecific antibodies were obtained from Cell Signaling Technology (Beverly, MA). Recombinant PDGF and neutralizing PDGF antibodies were purchased from R&D Systems (Minneapolis, MN). Horseradish peroxidase (HRP)-conjugated IgG antibody (Santa Cruz Biotechnology, Santa Cruz, CA) was used as the secondary antibody.

### Cell culture and mechanical stretch

Primary VSMC was obtained from the aorta of Sprague-Dawley rats (Charles River Breeding Laboratories, Kingston, NY, USA). Briefly, the aorta was dissected, cut into ∼1 mm^2^ segments, and then placed as explants in cell culture dishes containing DMEM (Gibco BRL, Grand Island, NY) with 10% FBS. VSMC purity was determined by staining with smooth-muscle-specific actin monoclonal antibodies (Sigma, St Louis, MO). To apply MS on VSMC, cells (6×10^5^ cells/well) were seeded onto 6-well Bioflex® plates (Dunn Labortechnik, Germany), which contain a pronectin-coated silicon membrane bottom. When cells reached confluency, media were replaced with serum-free media and cells were exposed to MS. A Flexercell® Tension Plus FX-4000T system (Flexcell International Corp. Hillsborough, NC) was used to apply physiological equibiaxial cyclic stretch (1 Hz, 0–10% strain, 60 cycles/min, 0–12 hrs).

### Immunofluorescence analysis

VSMC was fixed with 4% paraformaldehyde, and permeabilized with 50 mM NH_4_CL_3_ and 0.2% Triton X-100. After non-specific binding sites were blocked with 10% normal donkey serum, cells were incubated with specific primary antibodies. Cells were washed with 0.2% Triton X-100 in PBS, and then incubated with Cy3-conjugated IgG (Zymed Laboratries; Invitrogen, Carlsbad, CA). The stained cells were mounted in carbonate-buffered glycerol, and evaluated using a laser scanning confocal microscope (LSM 510, Carl Zeiss Inc., Germany).

### Cell viability assay

The MTT assay was used to determine the viability of VSMC. The assay measures the ability of an active mitochondrial enzyme to reduce the MTT substrate (yellow to blue) in live cells. Briefly, MTT working solution was added to each well, and after incubation at 37°C for 4 hrs the MTT solution was removed and 100 ml of dimethyl sulfoxide (DMSO) was added to dissolve the dark purple water-insoluble crystals. OD values obtained at a wavelength of 570 nm were subtracted from the values obtained at 630 nm to standardize the different measurements. Relative proliferation rates were determined by comparing strained cells with static control cells.

### Measurement of ROS

Changes in intracellular ROS levels were evaluated by measuring the oxidative conversion of DCFH-DA (Molecular Probes; Eugene, OR, USA) to fluorescent DCF. The cells grown in 6-well Bioflex® plates were incubated with 10 μM DCFH-DA for 30 min at 37°C, and then incubated with 10% MS for 10 min. After incubation, the cells were washed with PBS and then the fluorescence of DCF was detected using an Axiovert 200 fluorescence microscope (Carl Zeiss; Oberkochen, Germany). Fluorescence intensity was quantified using a Metamorph image analysis system (Molecular Devices, LLC, Downingtown, PA, USA).

### Measurement of MMP-2 promoter activity

The 5′-flanking promoter region from mouse genomic DNA was amplified by PCR using upstream primer 5′-AAGGTG*GCTAGC*TCCGTAACGTAGTAG-3′ and downstream primer 5′-ATCTAA*AGATCT*GGATGCACACAGAGC-3′; the *Nhe*I and *Bgl*II restriction enzyme sites are in italic. Both primers were designed on the basis of a sequence retrieved from GenBank Accession Nos. NM008610 and BC070430. The amplified 1584-bp fragment was cloned into pGL3 Basic vector (pGL3-MMP-2). The identity of the resulting constructs was verified by restriction enzyme digestion and sequence analysis.

pGL3-MMP-2 luciferase reporter plasmid DNA was prepared using QIAprep Spin Miniprep Kit (Qiagen, Valencia, CA, USA). After cells were transiently transfected with MMP-2 luciferase reporter plasmids using Lipofectamine 2000 (Invitrogen), luciferase activity in cell lysates was determined by a dual luciferase reporter assay system using a Glomax 20/20 luminometer (Promega).

### Measurement of mRNA expression

The expression of MMP-2 mRNA in VSMC was quantified by RT-PCR analysis, using GAPDH mRNA as an internal standard. Total RNA in cultured cells was isolated using Trizol reagent (Invitrogen, San Diego, CA, USA) and was reverse transcribed into cDNA using the Improm-II Reverse Transcription System (Promega). Amplification of cDNA by PCR was performed using the specific primers for MMP-2 (forward, 5′-GTCTTCCCCTTCACTTTTCTG-3′; reverse, 5′-CGGAAGTTCTTGGTGTAGGTG-3′).

### Immunoblot analysis

Cell lysates were prepared from cultured VSMC in ice-cold lysis buffer. Equal amounts of the lysates were separated on 8–10% SDS-polyacrylamide gel under reducing conditions and then transferred onto nitrocellulose membranes (Amersham-Pharmacia Biotech, Piscataway, NJ). Membranes were blocked for 2 hrs at room temperature in 5% skim milk in TBST and then incubated overnight with primary antibody in 3% BSA. Blots were washed with TBST and incubated 1 hr at room temperature with the HRP-conjugated secondary antibody. Blots were developed in the ECL Western blot detection reagents (Amersham-Pharmacia Biotech, Piscataway, NJ, USA). This membrane was re-blotted with anti-β-Actin antibody (HP Bromedicals, Aurora, Ohio) as an internal control.

### Gelatin zymography

To assess gelatinase activity, the extracellular medium from cultured VSMC was collected and concentrated 30-fold using a Vivaspin 2 centricon (Sartorius Biolab products, Sartorius AG). The concentrated medium was electrophoretically separated on 8% SDS-polyacrylamide gel containing 0.15% gelatin (Sigma). After electrophoresis, the gel was washed with 2.5% of Triton-containing wash buffer, activated in a 37°C incubator and then stained with 0.2% Coomassie brilliant blue R-250 (Sigma). Clear zones against the blue background indicated gelatinolytic activity.

### Transfection of siRNA

Small interfering RNA (siRNA) for Akt and PDGFR was designed and synthesized using a Silencer^TM^siRNA construction kit from Ambion (Austin, TX). For siRNA transfection, VSMC was seeded in 6-well plates and grown for 24 hrs. The cells were transfected with siRNA for Akt or PDGFR or a scrambled siRNA (negative control) using Lipofectamine 2000 (Invitrogen, CA), according to the manufacturer's instructions. Transfection efficiencies were monitored using a fluorescent oligonucleotide (BLOCK-iT Fluorescent Oligo; Invitrogen), and were estimated to be ∼80 to 90%.

### Statistical Analysis

All data were expressed as means ± SEM. The change in variable parameters between untreated control and treated groups was analyzed by one-way analysis of variance (ANOVA) followed by Tukey's multiple comparison tests as a post hoc comparison. Differences in parameters were considered statistically significant at *p*<0.05.

## Results

### MS enhances MMP-2 activity and production in VSMC

**Figure 1:**
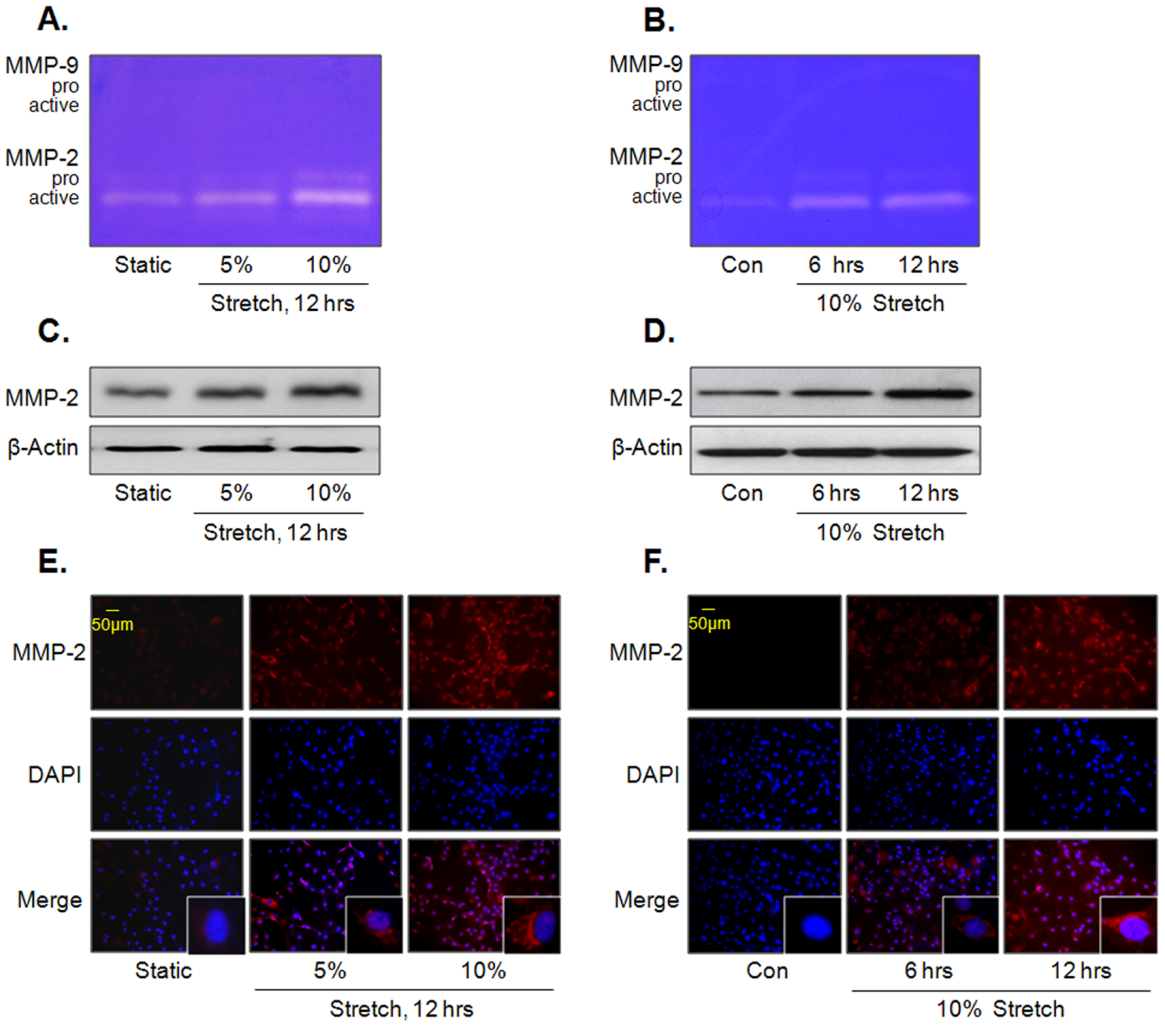
Effects of MS on MMP-2 activity and expression in VSMC. Cells were stimulated by MS at indicated forces (A) or time (B), and then gelatinolytic activity (MMP-2 and -9) was determined using gelatin zymography. The force- and time-dependent increase in MMP-2 expression in VSMC exposed to MS was determined by Western blot (C and D) and immunocytochemical studies (E and F). Representative images are from 4–6 independent experiments.

MMP activity was measured using extracts prepared from culture media of primary VSMC exposed to MS. Gelatin zymography showed that MS increased MMP-2 activity, but not MMP-9, in force (5 and 10%)- and time (6 and 12 hrs)-dependent manners (**Figure 1A and 1B**). In line with these results, the force- and time-dependent increase in cellular MMP-2 expression was demonstrated by Western blot analysis (**Figure 1C** **and1D**) as well as by immunocytochemical studies (**Figure 1E and 1F**).

### Involvement of Akt pathway in MS-induced MMP-2 production

**Figure 2:**
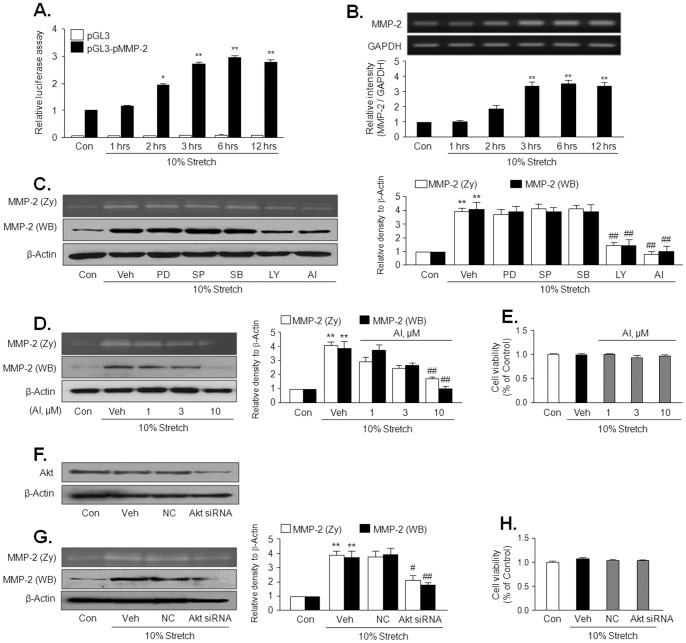
Role of Akt in MS-induced MMP-2 production in VSMC. (A) Time-course of MS-enhanced MMP-2 promoter activity. VSMC was transfected with MMP-2 promoter-luciferase construct (pGL-MMP-2) or empty luciferase vector (pGL3) for 24 hrs and then stimulated with 10% MS for the indicated time. MMP-2 promoter activity was represented as relative luciferase activity. (B) Time-course of MS-induced MMP-2 mRNA expression. Quantitative data are presented as the means ± SEM (n = 5–6). **p<0.01, *vs.* control (Con). (C and D) Cells were stimulated by10% MS for 12 hrs in the absence (Veh) or presence of various inhibitors including a PI3K inhibitor, LY294002 (LY, 10 μM), Akt inhibitor IV (AI, 10 μM), and MAPK inhibitors, PD98059 (PD, 10 μM), SP600125 (SP, 10 μM) and SB203580 (SB, 10 μM). (F and G) Cells were transfected with scrambled (NC, negative control) and AktsiRNA (100 pmol), and then stimulated by 10% MS for 12 hrs. MMP-2 activity and expression were measured using gelatin zymography and Western blot, respectively. Right panels represent quantitative data for left panels, and represent means ± SEM (n = 4). **p<0.01 *vs.* corresponding control (Con). #*p*<0.05, ##*p*<0.01 *vs.* corresponding vehicle (Veh). (E and H) Cell viability in VSMC treated with Akt inhibitor or transfected with Akt siRNA are represented as % of control, and data are presented as the means ± SEM (n = 4).

To investigate the MMP-2 promoter activity in VSMC stimulated by 10% MS, the MMP-2 promoter construct were transfected into cells, and then the reporter activity was measured. The MMP-2 promoter activity in 10% MS-stimulated cells was started to increase at 2 hrs, and remained high level until 12 hrs after 10% MS (**Figure 2A**). Similarly, MMP-2 mRNA expression was also started to increase at 2 hrs, and significantly increased after 3 hrs of 10% MS (**Figure 2B**). These results suggest that the increased in MMP-2 expression at 6 hrs and 12 hrs after 10% MS might be regulated at the transcriptional levels.

To investigate the signaling pathways involved in MS-induced MMP-2 production, VSMC was treated with 10% MS for 12 hrs in the presence or absence of pharmacological inhibitors for various MAPKs and PI3K/Akt pathways, such as PD98059 (an ERK inhibitor), SB203580 (a P38MAPK inhibitor), SP600125 (a JNK inhibitor), LY394002 (a PI3K inhibitor), and AI (an Akt inhibitor). As shown in **Figure 2C and 2D**, 10% MS-induced increases in MMP-2 activity and expression were attenuated by inhibitors for PI3K and Akt, but not by other MAPK inhibitors, as well as by molecular inhibition of Akt using Akt siRNA (**Figure 2Fand 2G**). These results suggest a pivotal role for the Akt pathway in MS-induced MMP-2 production in VSMC.

### PDGFR mediates Akt phosphorylation induced by MS

**Figure 3:**
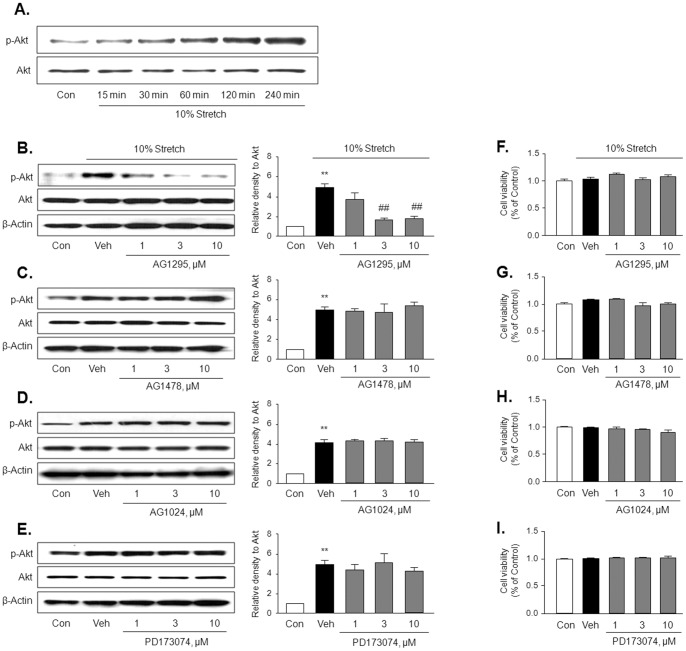
Role of PDGF receptor in MS-induced Akt phosphorylation in VSMC. (A) Representative Western blots for the phosphorylated Akt (p-Akt) and total Akt (Akt) in cells stimulated by 10% MS at the indicated time (n = 5). (B–E) Representative Western blots for p-Akt and Akt in cells stimulated by 10% MS for 4 hrs in the absence (Veh) or presence of inhibitors for various growth factor receptors, including AG1295 (a PDGF receptor inhibitor), AG1478 (an EGF receptor inhibitor), AG1024 (an IGF receptor inhibitor), and PD173074 (a FGF receptor inhibitor). Right panels represent quantitative data for the corresponding blots in the left panels, and data are presented as means ± SEM (n = 6). **p<0.01 *vs.* control (Con). ##*p*<0.01 *vs.* vehicle (Veh). (F–I) Cell viability in VSMC treated with various inhibitors are represented as % of control, and data are presented as the means ± SEM (n = 4).

Akt phosphorylation at Ser473 in 10% MS-stimulated VSMC was increased in a time-dependent manner up to 4 hrs (**Figure 3A**), suggesting that mechanoreceptors on the cellular membrane link mechanical stress and Akt. Because receptors for growth factors are known to transmit signals by mechanical stress [22], and EGF receptor transactivation induces activation of PI3K/Akt pathway [23], VSMC was treated with 10% MS for 4 hrs in the presence of inhibitors for various growth factor receptors, including AG1295 (a PDGFR inhibitor), AG1478 (an EGFR inhibitor), AG1024 (an IGFR inhibitor) and PD173074 (a FGFR inhibitor). Then Akt phosphorylation at Ser473 was assessed by immunoblotting. As shown in **Figure 3B–3E**, Akt phosphorylation induced by MS was inhibited by a PDGFR inhibitor in a dose-dependent manner, but not by other inhibitors of EGF, IGF and FGF receptors. These results suggest a central role for the PDGF receptor in conveying extracellular mechanical signals to the intracellular Akt pathway.

### PDGFR activation in response to MS

**Figure 4:**
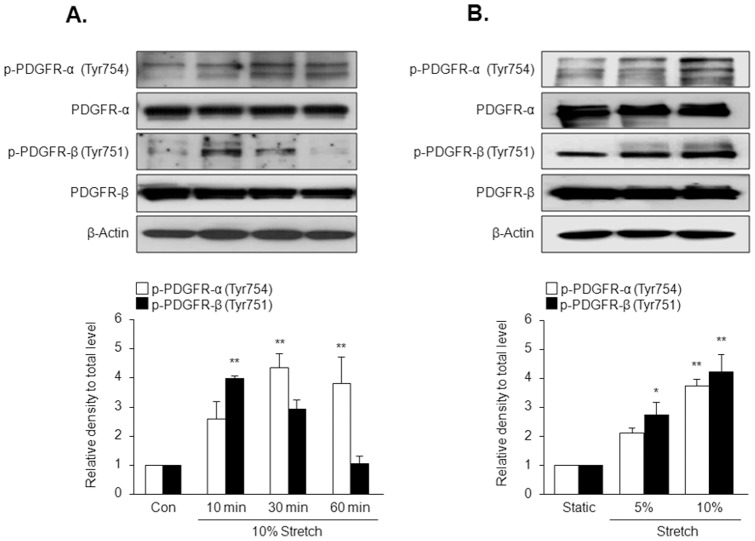
Time-course and force-dependent effects of MS on the phosphorylation of PDGFR isoforms in VSMC. Cells were stimulated by 10% MS for the indicated time (A), or stimulated with different forces of MS for 10 min (B), and then the levels of phosphorylated and total PDGFR isoforms (PDGFR-α and PDGFR-β) were determined by immunoblotting. Bottom panels represent quantitative data for the corresponding blots in the upper panels, and data are presented as means ± SEM (n = 5). *p<0.05, **p<0.01 *vs.* corresponding control (Con).

To obtain direct evidence that physical forces induce PDGFR activation, phosphorylation of both PDGFR-α (Tyr754) and PDGFR-β (Tyr751) was examined by immunobloting with specific antibodies. Phosphorylation of PDGFR-α and PDGFR-β in 10% MS-stimulated cells was increased as early as 10 min (**Figure 4A**). Maximal phosphorylation of PDGFR-α and PDGFR-β was achieved 30 min and 10 min after 10% MS, respectively. To further study the effect of MS on PDGFR phosphorylation, VSMC was stretched for elongations of 5 and 10% of original size, and then phosphorylation of PDGFR-α and PDGFR-β was assessed. As shown in **Figure 4B**, the magnitudes of phosphorylation of PDGFR-α and PDGFR-β were higher in VSMC exposed to 10% MS than in VSMC exposed to 5% elongation, indicating that a certain level of mechanical force is needed for PDGFR phosphorylation.

### Involvement of ROS in MS-induced phosphorylation of PDGFR

**Figure 5:**
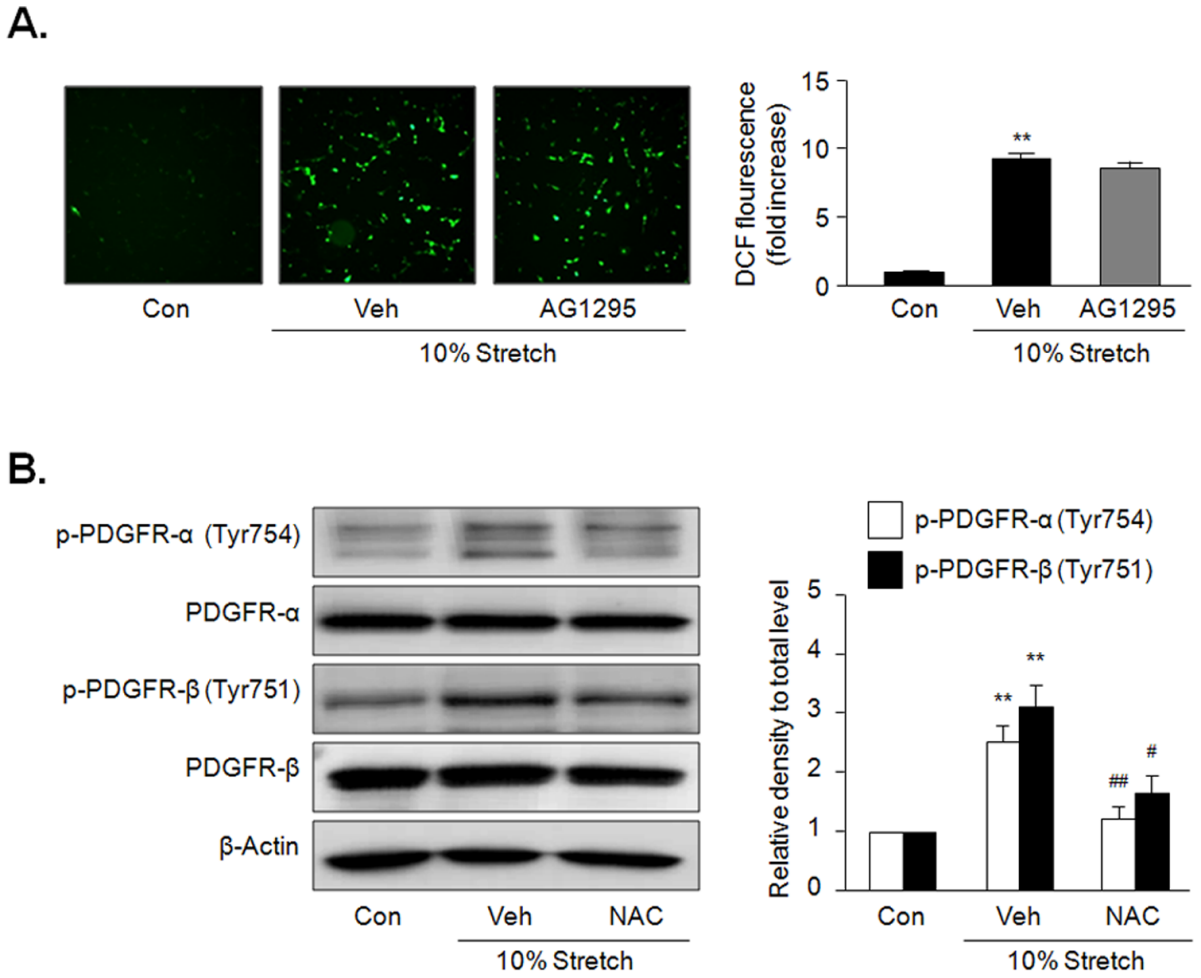
Involvement of ROS in MS-induced PDGFR phosphorylation. (A) Cells were stimulated by 10% MS for 10 min, and ROS was determined using DCF fluorescence. Quantitative data for the corresponding DCF fluorescence in the left are presented as means ± SEM (n = 4). **p<0.01 *vs.* control (Con). (B) Cells were stimulated by 10% MS for 10 min in the presence or absence of NAC (0.5 mM), a ROS inhibitor, and the levels of phosphorylated and total PDGFR isoforms (PDGFR-α and PDGFR-β) were determined by immunoblotting. Quantitative data for the corresponding blots in the left panel are presented as means ± SEM (n = 5). **p<0.01 *vs.* corresponding control (Con). #*p*<0.05, ##*p*<0.01 *vs.* corresponding vehicle (Veh).

To investigate the potential involvement of ROS in MS-induced activation of PDGFR, we determined ROS in VSMC stimulated by 10% MS. As shown in **Figure 5A**, ROS production measured by DCF fluorescence was markedly increased in VSMC stimulated by 10% MS for 10 min, which was not affected by AG1295, a PDGFR inhibitor. In contrast, the increased phosphorylation of PDGFR-α and PDGFR-β in cells stimulated by 10% MS was significantly attenuated in cells pretreated with NAC, a ROS inhibitor (**Figure 5B**), suggesting a potential role of ROS in MS-induced phosphorylation of PDGFR.

### PDGFR-β links MS and Akt phosphorylation

**Figure 6:**
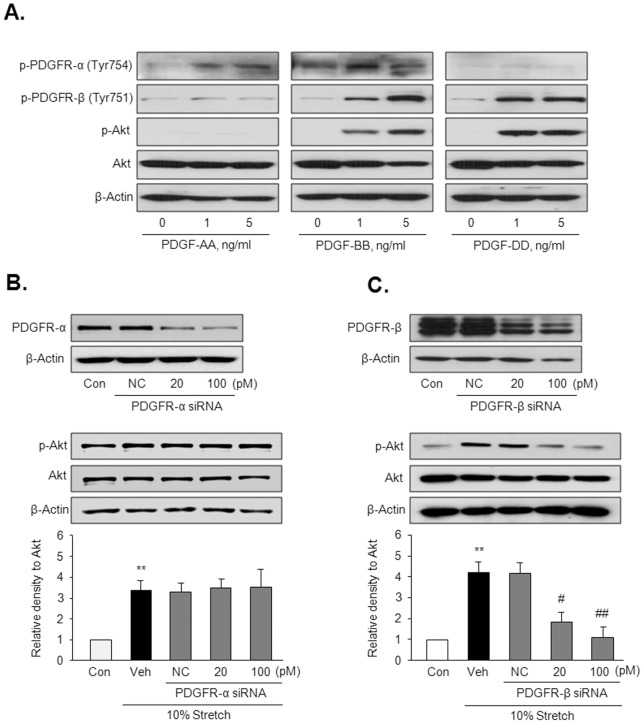
The individual roles of PDGFR isoforms in Akt phosphorylation in VSMC. (A) Representative immunoblots for the phosphorylated (p-Akt) and total Akt in cells stimulated with a PDGFR-α ligand (PDGF-AA) and PDGFR-β ligands (PDGF-BB and –DD) for 10 min (n = 5). (B) Cells were transfected with the indicated doses of PDGFR-α siRNA or PDGFR-β siRNA, and then stimulated by 10% MS for 4 hrs. Quantitative data for the corresponding blots are presented as means ± SEM (n = 5). **p<0.01 *vs.* control (Con). #*p*<0.05, ##*p*<0.01 *vs.* vehicle (Veh).

To evaluate the individual role of PDGFR isoforms in Akt phosphorylation in response to MS, Akt phosphorylation was determined in VSMC stimulated with ligands for PDGFR-α and PDGFR-β. As shown in **Figure 6A**, PDGFR-β ligands including PDGF-BB and -DD increased Akt phosphorylation, whereas PDGF-AA, a PDGFR-α ligand, had no effect on Akt phosphorylation in VSMC. To further determine the individual role of PDGFR-α and PDGFR-β in MS-induced Akt phosphorylation, PDGFR-α and PDGFR-β were depleted in VSMC using PDGFR-α siRNA and PDGFR-β siRNA, respectively. VSMC was then exposed to 10% MS for 4 hrs. As expected, Akt phosphorylation induced by 10% MS was markedly attenuated by molecular inhibition of PDGFR-β, but not by inhibition of PDGFR-α (**Figure 6B and 6C**), indicating a central role for PDGFR-β in MS-induced Akt activation.

### Role of PDGFR-β in mechanical stress-induced MMP-2 production

**Figure 7:**
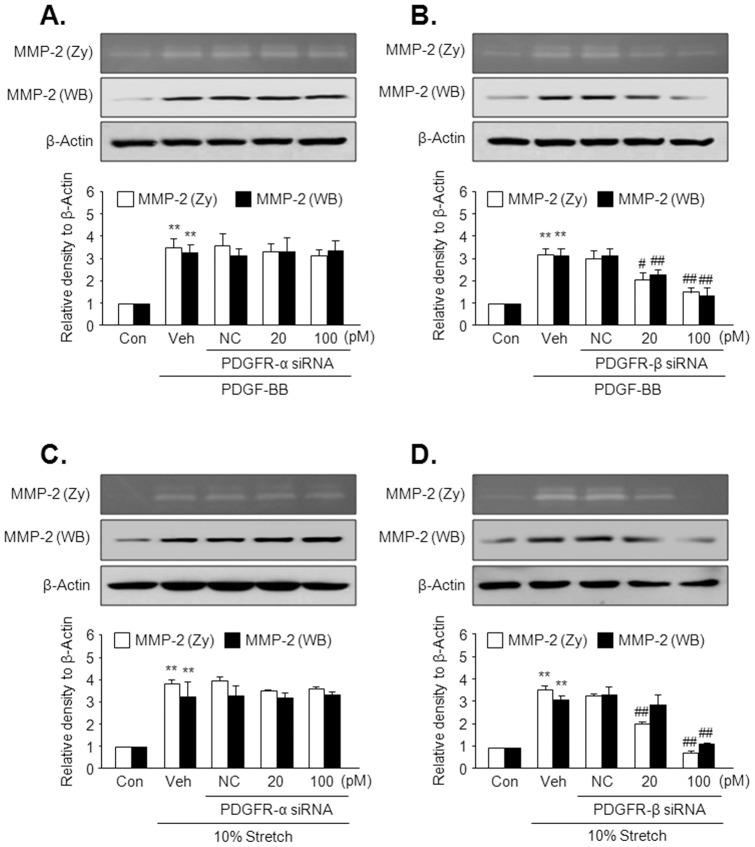
The individual roles of PDGFR isoforms in MS-induced MMP-2 production in VSMC. Cells were transfected with the indicated doses of PDGFR-α siRNA or PDGFR-β siRNA, and then stimulated with 5 ng/ml PDGF-BB (A and B) as well as by 10% MS (C and D) for 12 hrs. MMP-2 activity and production were determined using gelatin zymography and Western blot, respectively. Quantitative data for the corresponding blots are presented as means ± SEM (n = 6). **p<0.01 *vs.* corresponding control (Con). #*p*<0.05, ##*p*<0.01 *vs.* corresponding vehicle (Veh).

To investigate the individual roles for PDGFR-α and PDGFR-β in MMP-2 production, the effects of PDGF-BB or MS on MMP-2 production were determined using PDGFR-α or PDGFR-β-deficient cells. As shown in **Figure 7A and 7B**, PDGF-BB-induced increases in the MMP-2 production and activity were attenuated by molecular inhibition of PDGFR-β in VSMC, but not by inhibition of PDGFR-α. Likewise, the increased production and activity in VSMC stimulated by MS were attenuated by molecular inhibition of PDGFR-β in cells, but not by inhibition of PDGFR-α (**Figure 7C and 7D**).

## Discussion

In this study, we identified mechanical stretch (MS)-dependent signaling pathways that result in the enhanced expression of MMP-2 in VSMC. This study provided evidences to support a functional role for MS in the regulation of PDGF receptor (PDGFR) activity, which subsequently activates the Akt signaling pathway. Although both PDGFR-α and PDGFR-β were activated by MS, the increase in Akt phosphorylation in VSMC exposed to MS was mediated by PDGFR-β, but not PDGFR-α. Thus, MS-induced MMP-2 production in VSMC appears to be mediated via activation of the PDGFR-β-Akt signaling axis.

Increased blood pressure, leading to mechanical stress on VSMC in the medial layer of the vasculature, is an important stimulus that induces vascular remodeling [1], [19]. However, the underlying mechanisms linking hypertension with vascular remodeling are unknown. Because MMP plays a key role in tissue remodeling associated with vascular lesion progression [24], this study investigated the expression of gelatinases in VSMC exposed to MS. Consistent with previous studies in which MS increased MMP-2 expression in VSMC [25] and atrial myocytes [26], our results showed that MMP-2 expression and secretion, but not MMP-9, were increased in VSMC exposed to 5 and 10% MS. This suggests a potential role for MMP-2 in hypertension-related vascular remodeling. Moreover, the magnitudes of MMP-2 production and secretion in VSMC exposed to 10% MS were higher than those in VSMC exposed to 5% elongation, indicating that a certain level of mechanical force is needed for MMP-2 production with subsequent vascular remodeling.

MMP-2 transcription is induced through the PI3K/Akt pathway and this pathway is necessary and sufficient for MMP-2 up-regulation in VSMC [27]. Our previous studies have also shown that the PI3K/Akt pathway is critically involved in HNE-induced MMP-2 transcription in VSMC through activation of NF-κB [12]. Consistent with these previous reports, the MS-induced increases in MMP-2 activity and expression were attenuated by inhibitors for PI3K and Akt, but not by other MAPK inhibitors, as well as by molecular inhibition of Akt using Akt siRNA. In addition, MS increased phosphorylation of Akt in VSMC, and inhibition of the Akt pathway attenuated MMP-2 expression stimulated by MS. These results implicate the activation of the PI3K/Akt pathway in response to MS for the up-regulation of MMP-2 expression and secretion in VSMC.

Receptors for growth factors are known to transmit signals by stimuli other than ligand binding, including mechanical stress [14], [17]. Recently, several membrane proteins including integrins and receptor tyrosine kinases such as receptors for PDGF, EGF, IGF and FGF have been shown to be mechanosensitive [20]. As intracellular mechanosensors for growth factor signaling, the importance of Akt pathways has been demonstrated in mesangial cells [28], epithelial cells [29] and VSMC [13], [30]. In line with these previous studies, our present data from pharmacological inhibitors showed that PDGFR inhibition attenuated Akt activation induced by mechanical stress, suggesting cross-talk between PDGFR and Akt in VSMC exposed to MS. However, in contrast to the previous study describing the important role of other receptors for growth factors including EGF in MS-mediated signaling axis [31], MS-induced Akt phosphorylation was not inhibited by inhibitors for EGFR, IGFR and FGFR in VSMC in the present study. At present, we cannot explain why PDGFR, but not EGFR, IGFR and FGFR, was exclusively involved in Akt phosphorylation in VSMC. Considering the existence of differential responses to MS between cell types, the upstream events regulating Akt phosphorylation are likely dependent on cell types as well as stress types.

Although numerous studies have described the downstream targets of PDGF that modulate VSMC phenotype [32], [33], there is a dearth of knowledge regarding PDGF-stimulated mechanisms in vascular remodeling. Previous report has described the increases in the level of PDGF and its receptors in mechanically stimulated tissues [34]. Wilson et al. (1993) reported an increase in PDGF-AA and -BB production by neonatal rat VSMC subjected to MS and demonstrated autocrine stimulation by secreted PDGF [35]. In contrast, Shimizu et al. (2008) observed rapid phosphorylation of the PDGFR in VSMC subjected to cyclic stretch that could not be blocked by PDGF-neutralizing antibody. In line with previous reports in which physical forces have been implicated in ligand-independent activation of PDGFR [16], [17], our data also showed that both PDGFR-α and PDGFR-β were activated by MS, which was not inhibited by neutralizing antibodies that bind to all forms of PDGF (data not shown), suggesting a ligand-independent activation of PDGFR.

In the present study, MS-stimulated phosphorylation of PDGFR-α and PDGFR-β was observed as early as 10 min. Maximal phosphorylation of PDGFR-α and PDGFR-β was achieved 30 min and 10 min after MS, respectively, and returned to baseline by 60 min. Reportedly, PDGFR activation increased intracellular ROS production [36], and MS increased PDGFR phosphorylation [37], suggesting a potential role of PDGFR in MS-induced ROS generation. However, while MS produced ROS production as early as 1–5 min in VSMC [11], PDGFR phosphorylation was evident at 8 min after MS [37]. In addition, MS-induced ROS production was not inhibited by PDGFR inhibitor in our present study, suggesting a negligible role of PDGFR in MS-induced ROS generation in VSMC. In contrast, in line with previous data in which ROS mediates PDGFR phophorylation in VSMC [38], the increased phosphorylation of PDGFR-α and PDGFR-β in cells stimulated by 10% MS was significantly attenuated by pretreatment with NAC, a ROS inhibitor, suggesting a potential role of ROS in MS-induced phosphorylation of PDGFR. To further study the effect of mechanical stress on PDGFR phosphorylation, VSMC was stretched for elongations of 5 and 10% of the original size, and then phosphorylation of PDGFR-α and PDGFR-β in protein extracts were determined. The magnitudes of phosphorylation of PDGFR-α and PDGFR-β were higher in VSMC exposed to 10% stretch than in VSMC exposed to 5% elongation, indicating that a certain level of mechanical force is needed for PDGFR phosphorylation.

Because the individual roles of PDGFR-α and PDGFR-β are independent in VSMC development [39], we tried to identify the individual role of PDGFR isoforms on Akt phosphorylation in response to MS. Consistent with a previous report describing a critical role for PDGFR-β in PI3K/Akt signaling in mesenchymal stem cells [40], PDGFR-β ligands including PDGF-BB and -DD increased Akt phosphorylation, whereas PDGF-AA, a PDGFR-α ligand, had no effect on Akt phosphorylation in VSMC that were not exposed to MS. Considering that transactivation of EGFR by PDGF-BB was not observed in arterial VSMC [19], our data suggest that PDGFR-β might play a potential role in Akt phosphorylation in VSMC exposed to MS. To further determine the individual role of PDGFR subtypes in MS-induced Akt phosphorylation, cells were exposed to 5 and 10% MS for 4 hrs after individual deletion of PDGFR using the respective siRNA. As expected from another report in which the PDGFR-β signaling axis was involved in phenotypic modulation of VSMC [41], although both PDGFR-α and PDGFR-β were activated by MS, inhibition of PDGFR-β with siRNA, but not PDGFR-α, attenuated MMP-2 production as well as Akt phosphorylation mediated by MS.

Taken together, it is concluded that MS induces MMP-2 production in VSMC via PDGFR-β-dependent activation of Akt pathway. These findings suggest a novel role for the PDGFR-β/Akt signaling axis in the progression of vascular diseases induced by hypertension.

## Conclusions

Our present study demonstrated that PDGFR-β, as a cell surface mechanoreceptor, conveys mechanical signals to intracellular sensors to produce MMP-2 via regulation of Akt activity in VSMC exposed to MS, suggesting that PDGFR-β/Akt signaling axis might play a pivotal role in vascular remodeling induced by mechanical stress linked to arterial hypertension.
